# Identification of hub genes associated with the pathogenesis of diffuse large B-cell lymphoma subtype one characterized by host response via integrated bioinformatic analyses

**DOI:** 10.7717/peerj.10269

**Published:** 2020-11-20

**Authors:** Lingna Zhou, Liya Ding, Yuqi Gong, Jing Zhao, Gong Xin, Ren Zhou, Wei Zhang

**Affiliations:** 1 Department of Pathology and Physiology, Cancer Institute, Second Affiliated Hospital, Zhejiang University School of Medicine, Hangzhou, Zhejiang, China; 2 Department of Pathology, The First Affiliated Hospital, Zhejiang University School of Medicine, Hangzhou, Zhejiang, China; 3 Department of Pathology and Pathophysiology, Institute of Pathology and Forensic Medicine, Zhejiang University School of Medicine, Hangzhou, Zhejiang, China; 4 Key Laboratory of Disease Proteomics of Zhejiang Province, Zhejiang University School of Medicine, Hangzhou, Zhejiang, China; 5 School of Life Sciences, Westlake University, Hangzhou, Zhejiang, China, Hangzhou, Zhejiang, China

**Keywords:** Host response (HR), Diffuse large B-cell lymphoma (DLBCL), Weighted geneco-expression network analysis (WGCNA), Integrated bioinformatic analyses

## Abstract

**Background:**

Host response diffuse large B-cell lymphoma (HR DLBCL) shares features of histologically defined T-cell/histiocyte-rich B-cell lymphoma, including fewer genetic abnormalities, frequent splenic and bone marrow involvement, and younger age at presentation. HR DLBCL is inherently less responsive to the standard treatment for DLBCL. Moreover, the mechanism of infiltration of HR DLBCL with preexisting abundant T-cells and dendritic cells is unknown, and their associated underlying immune responses incompletely defined. Here, hub genes and pathogenesis associated with HR DLBCL were explored to reveal molecular mechanisms and treatment targets.

**Methods:**

Differentially expressed genes were identified in three datasets (GSE25638, GSE44337, GSE56315). The expression profile of the genes in the GSE53786 dataset was used to constructed a co-expression network. Protein-protein interactions analysis in the modules of interest identified candidate hub genes. Then screening of real hub genes was carried out by survival analysis within the GSE53786 and GSE10846 datasets. Expression of hub genes was validated in the Gene expression profiling interactive analysis, Oncomine databases and human tissue specimens. Functional enrichment analysis and Gene set enrichment analysis were utilized to investigate the potential mechanisms. Tumor Immune Estimation Resource and The Cancer Genome Atlas were used to mine the association of the hub gene with tumor immunity, potential upstream regulators were predicted using bioinformatics tools.

**Results:**

A total of 274 common differentially expressed genes were identified. Within the key module, we identified CXCL10 as a real hub gene. The validation of upregulated expression level of CXCL10 was consistent with our study. CXCL10 might have a regulatory effect on tumor immunity. The predicted miRNA (hsa-mir-6849-3p) and transcription factor (IRF9) might regulate gene expression in the hub module.

## Introduction

Diffuse large B-cell lymphoma (DLBCL) is the dominant subtype of non-Hodgkin lymphoma (Malumbres et al., 2008) and is characterized by clinical heterogeneity. This heterogeneity presents a challenge to the treatment of DLBCL. Although approximately 60% of the DLBCL patients achieved durable remission following the current standard chemotherapy R-CHOP (rituximab, cyclophosphamide, doxorubicin, vincristine, and prednisolone), 40% of patients suffered a relapse or became refractory, with limited treatment options (Cristino et al., 2019; Jiang et al., 2017).

Basing on gene expression profiling, a previous study classified patients into three subgroups: germinal center B-cell (GCB) group, activated B-cell group, and unclassified group (Alizadeh et al., 2000). The unclassified group indicated the molecular heterogeneity, which poses a barrier for better treatment and understanding of DLBCL. Thus, novel classification is urgently needed to better distinguish subtypes for DLBCL.

In 2005, Monti et al. (2005) identified three discrete DLBCL subtypes according to consensus clusters—“oxidative phosphorylation” (Oxphos) , “B-cell receptor/proliferation” (BCR), and “host response” (HR). HR DLBCL is T-cell/histiocyte-rich B-cell lymphoma, which suggests that tumor microenvironment may play an essential role in this subgroup of DLBCL. Tumor microenvironment has a closed relationship with the tumor initiation, progression, and invasion. Targeting the host immune response to HR DLBCL could be an effective treatment modality. However, the mechanism of infiltration of HR DLBCL with preexisting abundant T-cells and dendritic cells is unknown, and their associated underlying immune responses incompletely defined.

To further explore the mechanism of tumorigenesis and progression of HR DLBCL, we collected five published datasets of DLBCL from the Gene Expression Omnibus database (GEO), data from Gene Expression Profiling Interactive Analysis (GEPIA), Oncomine and The Cancer Genome Atlas (TCGA). Co-expression network analysis was applied to identify modules of common differentially expressed genes (DEGs) and determine how they correlated with HR DLBCL. To broadened our knowledge about the hub module and hub genes, comprehensive bioinformatics analyses were conducted, including functional enrichment analysis, gene set enrichment analysis, exploration of correlation of hub genes with tumor-infiltrating cells and immune signatures, upstream regulator prediction. By associating clinical data with molecular mechanisms, network-centric genes, new biomarkers for diagnosis, prognosis and treatment might be identified.

## Materials & Methods

### Data collection and preprocessing

**Figure 1:**
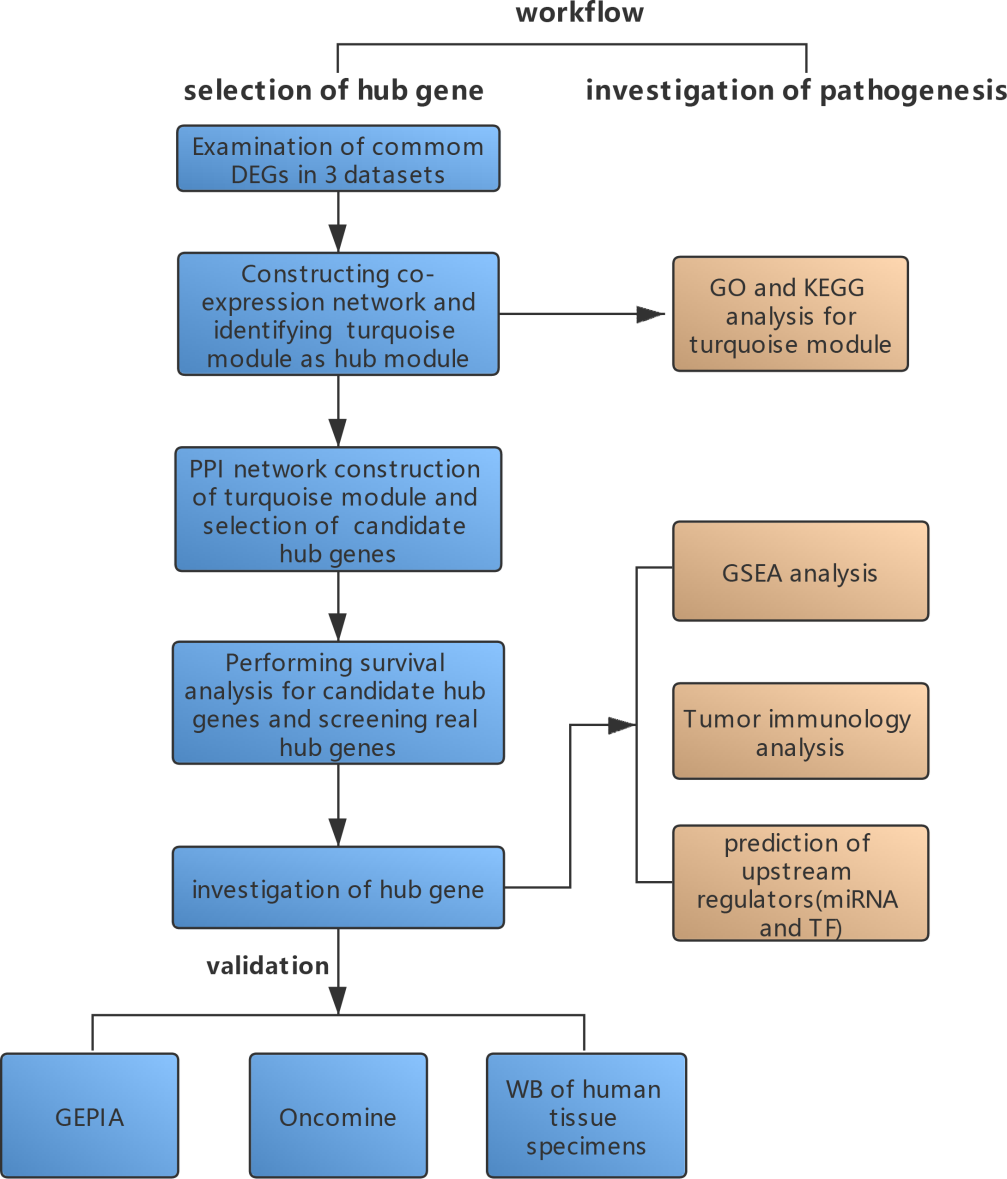
A schematic representation of the study methods. Methods regarding real hub gene selection and verification were shown in blue boxes, methods regarding molecular pathogenesis investigation were shown in orange boxes.

The workflow of public microarray repositories search is shown in Fig. S1. After a systematic search, five datasets (GSE25638, GSE44337, GSE56315, GSE53786 and GSE10846) depended on the GPL570 platform met the inclusion criteria and were included in the integrated bioinformatics analyses (Vicente-Duenas et al., 2012; Tompkins et al., 2013; Dybkaer et al., 2015; Scott et al., 2014; Cardesa-Salzmann et al., 2011). Normalized datasets were obtained from the GEO database using the GEOquery package (Davis & Meltzer, 2007). The maximum mean expression values of probes were used for genes with multiple probes in the microarray data and were annotated using the hgu133plus2.db. The clinical characteristics of included DLBCL patients were summarized in Table 1. The GSE25638, GSE44337 and GSE56315 datasets were analyzed for DEGs respectively. The intersections of the DEGs of the three datasets were selected for further analysis. GSE53786 dataset with typing data, survival, and prognosis information was used as the training set to construct co-expression networks. Then screening of real hub genes was carried out by survival analysis within the GSE53786 and GSE10846 datasets. A schematic representation of the study methods is shown in Fig. 1.

**Table 1:** Characteristics of the five studies inluded in the bioinformatic analysis.

Clinical features of included patients	*n* = 629
Platform	
GPL570[HG-U133_Plus_2]	629
Patient source	
USA	548
Spain	26
Denmark	55
Age	61.21764 ± 15.25502
Sex	
Male	292
Female	220
NA	117
Stage	
I	82
II	157
III	126
IV	153
NA	111

### Screening of DEGs

DEGs between DLBCL samples and normal tissues were identified through R package limma. DEGs with *P* < 0.05 and fold change (log FC) > Mean (log FC) + 2*SD (log FC) were regarded as significant DEGs.

### Co-expression network construction

Weighted gene co-expression network analysis (WGCNA) (Langfelder & Horvath, 2008) was applied to construct the co-expression network of overlap significant DEGs. Hierarchical clustering was carried out using the average method with exclusion by static tree cutting with a cut height set to 100. By calculating the scale-free fit index with a soft-threshold power (*β*) capability of 1 to 20, the one with the highest average connectivity degree was regarded as the most appropriate one to construct a scale-free network. Co-expressed genes were assigned to modules via dynamic minimum tree cutting, and similar modules were merged into one (similarity ≥ 0.75). We applied module eigengenes (MEs) and module gene significance (MS) to estimate correlations between clustered modules and the DLBCL subtypes.

### Candidate hub genes selection

All genes in the hub module were uploaded to Search Tool for the Retrieval of Interacting Genes (STRING) to predict protein-protein interactions(PPIs), then the PPIs were imported into Cytoscape for visualization (Szklarczyk et al., 2017; Zhou et al., 2018). To predict the important nodes, the plug-in CytoHubba was used to calculate the degree of genes in the hub module. We defined genes with node connectivity > ratio of total edges to total nodes as the candidate hub genes.

### Real hub gene identification

Expressions of candidate hub genes were divided into high and low levels using median expression level for further Kaplan–Meier survival analyses in GSE53786 and GSE10846.

Genes significantly correlated with prognosis (*P* < 0.05) were selected. Then, the prognosis values of the selected genes were assessed in different subgroups of DLBCL separately. The gene with significant prognosis value both in overall DLBCL patients and HR subtype was identified as real hub gene.

### Validation of expression level of the hub gene in DLBCL

The expression of the hub gene at the transcriptional level was displayed by Expression Profiling Interactive Analysis (GEPIA; http://gepia.cancer-pku.cn) (Tang et al., 2017) and Oncomine gene expression array database (http://www.oncomine.org) (Rhodes et al., 2004).

The expression of the real hub gene at the protein level was verified by western blot of human tissue specimens. All the human tissue specimens were obtained from the Department of Pathology, the First Affiliated Hospital, Zhejiang University School of Medicine, Hangzhou, China. Our study was approved by the Ethics Committee of Zhejiang University (Hangzhou, China). Under the guidance of the World Health Organization Classification of Tumors of Hematopoietic and Lymphoid Tissues, specimens, collected with informed consent, were diagnosed as DLBCL. The protein samples were eluted by boiling in 1 × SDS loading buffer at 100 °C and then fractionated by 12% SDS/PAGE, followed by standard western blotting. Antibodies were specific for anti-CXCL10 (Abcam, ab254374), anti-GAPDH (Abcam, ab181602).

### Functional enrichment analysis

Metascape (http://metascape.org/) integrates multiple databases, such as Gene Ontology (GO), Kyoyo Encyclopedia of Genes and Genomes (KEGG), UniProt, and DrugBank (Zhou et al., 2019). It provides the typical gene enrichment analysis and visualizes the results from lists of biological functions, pathways, and more (Ye et al., 2019). Functional enrichment analysis of the screened genes in the hub module was carried out by Metascape. *P* < 0.01 was set as the cutoff criterion, and significance was ranked by enrichment score (−log10(*P*-value)).

### Gene set enrichment analysis (GSEA)

Samples of HR DLBCL in GSE10846 were divided into two groups according to the expression level of the real hub gene. GSEA (version 4.1.0) was utilized to detect significantly different signaling in the gene rank between the two groups. False discovery rate (FDR) ≤ 0.25 and *p*-value ≤ 0.05 were recommended.

### Tumor immunology analysis

Tumor Immune Estimation Resource (TIMER, https://cistrome.shinyapps.io/timer/), containing 10,897 samples across 32 cancer types from TCGA (Li et al., 2017), provides a comprehensive analysis of immune infiltrates with tumors. We analyzed the correlation of the hub gene with various immune infiltrates in DLBCL, including B cells, CD8+ T cells, CD4+ T cells, macrophages, neutrophils, dendritic cells, and the tumor purity.

To explore the correlation of the hub gene with different kinds of immune signatures in DLBCL, we obtain gene signatures of various types of tumor-infiltrating lymphocytes as well as markers of chemokine, Major histocompatibility complex (MHC), Immunoinhibitor, Immunostimulatory, Cytokine and cytokine receptor, Immune checkpoint, Immune cell infiltrate genes, Cancer testis antigen genes, Human leukocyte antigen (HLA) genes, and Pro-inflammatory genes from the previous study (Liu et al., 2020). Besides, we downloaded data of patients with DLBCL mined from TCGA and RNA sequencing (RNA-seq) expression results using the RTCGA Toolbox package in R.

Correlations were calculated by the Pearson correlation and the threshold used for significant expression correlation was a Pearson correlation coefficient — r — ≥ 0.3 and all corresponding *P* values < 0.05.

### Construction of potential miRNA- and transcriptional factor (TF)-target regulatory networks

We used the miRNet to predict potential miRNAs in regulating genes in the hub module. The predicted miRNA with the highest was selected to construct miRNA-target networks. Transcription factors of the highly related module were analyzed by the iRegulon plugin in Cytoscape (Janky et al., 2014). TF-target network consisted of the predicted TF with the highest normalized enrichment score and targeted genes in the hub module.

## Results

### DEGs screening

**Figure 2:**
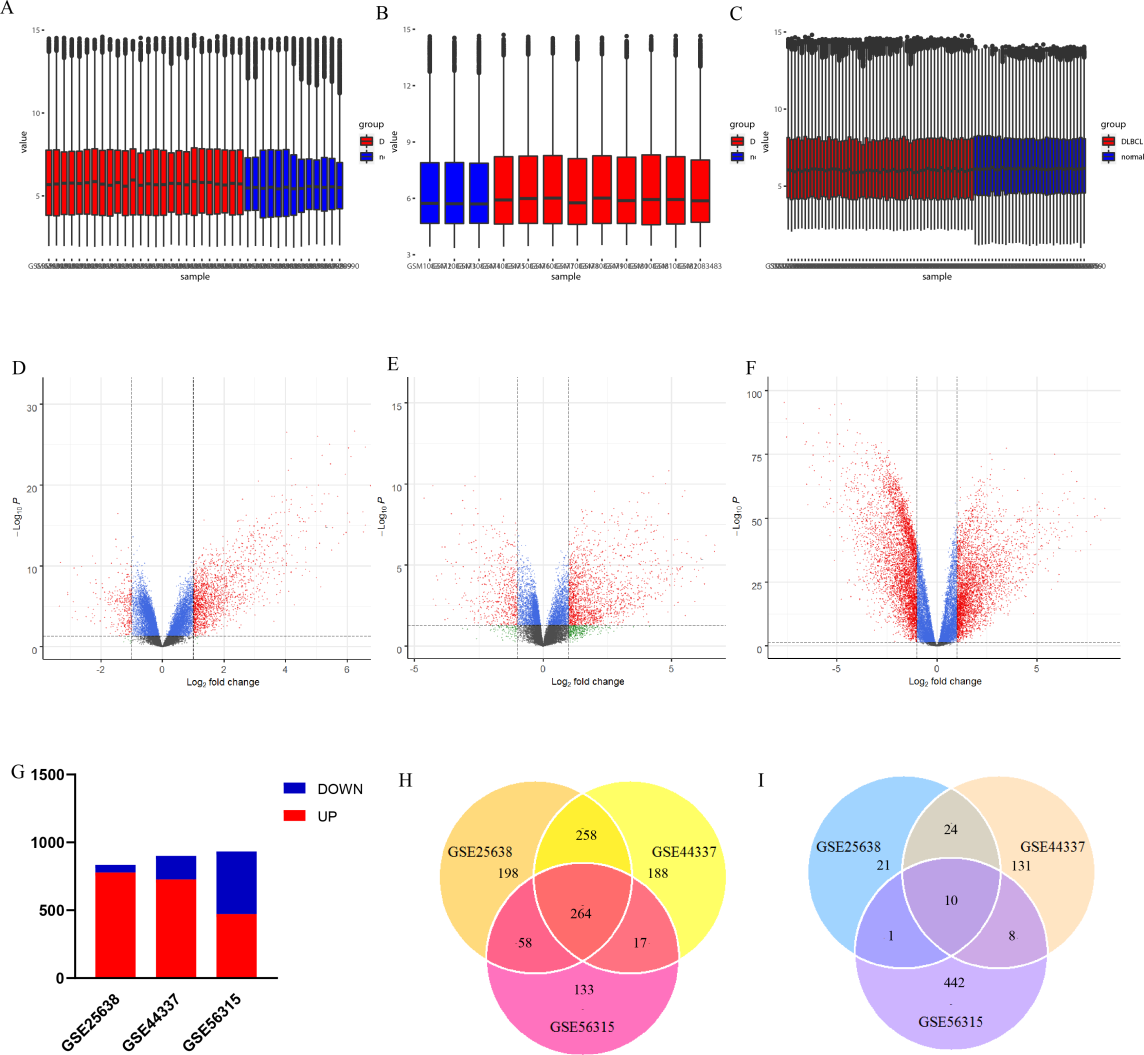
Statistics of differently expressed genes. (A–C) Box plots for the expression data in GSE25638, GSE44337 and GSE56315; (D–F) The volcano plots of DEGs in GSE25638, GSE44337, and GSE56315. The red dots represent upregulated DEGs while the green dots indicate downregulated DEGs; (G) DEGs in the three gene datasets; (H) Venn diagram analysis: overlapping upregulated differentially expressed genes from three datasets; (I) Venn diagram analysis: overlapping downregulated differentially expressed genes from three datasets.

After data preprocessing and quality assessment, normalized expression data of each dataset was shown by box plots (Figs. 2A–2C). Then DEGs were identified from each of the three datasets. Volcano plots were used to visualize DEGs between the tumor and non-tumor groups (Figs. 2D–2F). A total of 834 DEGs were screened from the GSE25638 dataset. They comprised 778 upregulated genes and 56 downregulated genes. Nine hundred DEGs were screened from the GSE44337 dataset. They comprised 727 upregulated genes and 173 downregulated genes. Nine hundred thirty-three DEGs were screened from the GSE56351 dataset, comprising 472 upregulated genes and 461 downregulated genes (Fig. 2G). A total of 274 common DEGs from the three datasets were selected for further analysis. These 274 DEGs comprised 264 upregulated genes (Fig. 2H) and ten downregulated genes (Fig. 2I).

### Construction of Weighted co-expression network and identification of the hub module

**Figure 3:**
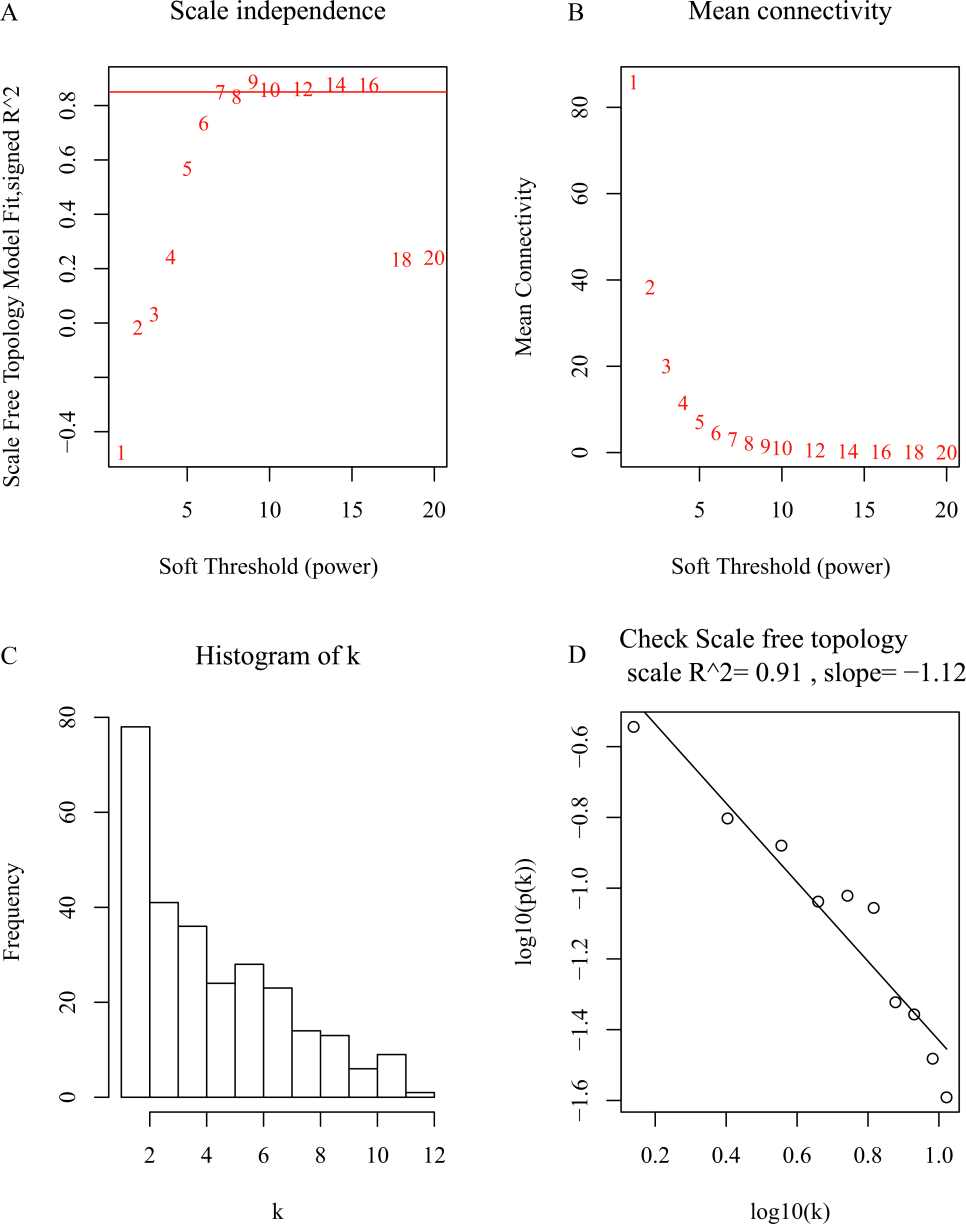
Soft-thresholding power selection in the weighted gene co-expression network analysis. (A) Scale-free fit index of various soft-thresholding powers (*β*); (B) mean connectivity of various soft-thresholding powers(*β*); (C) Histogram of the connectivity distribution when *β* = 7; (D) scale-free topology checking when *β* = 7.

**Figure 4:**
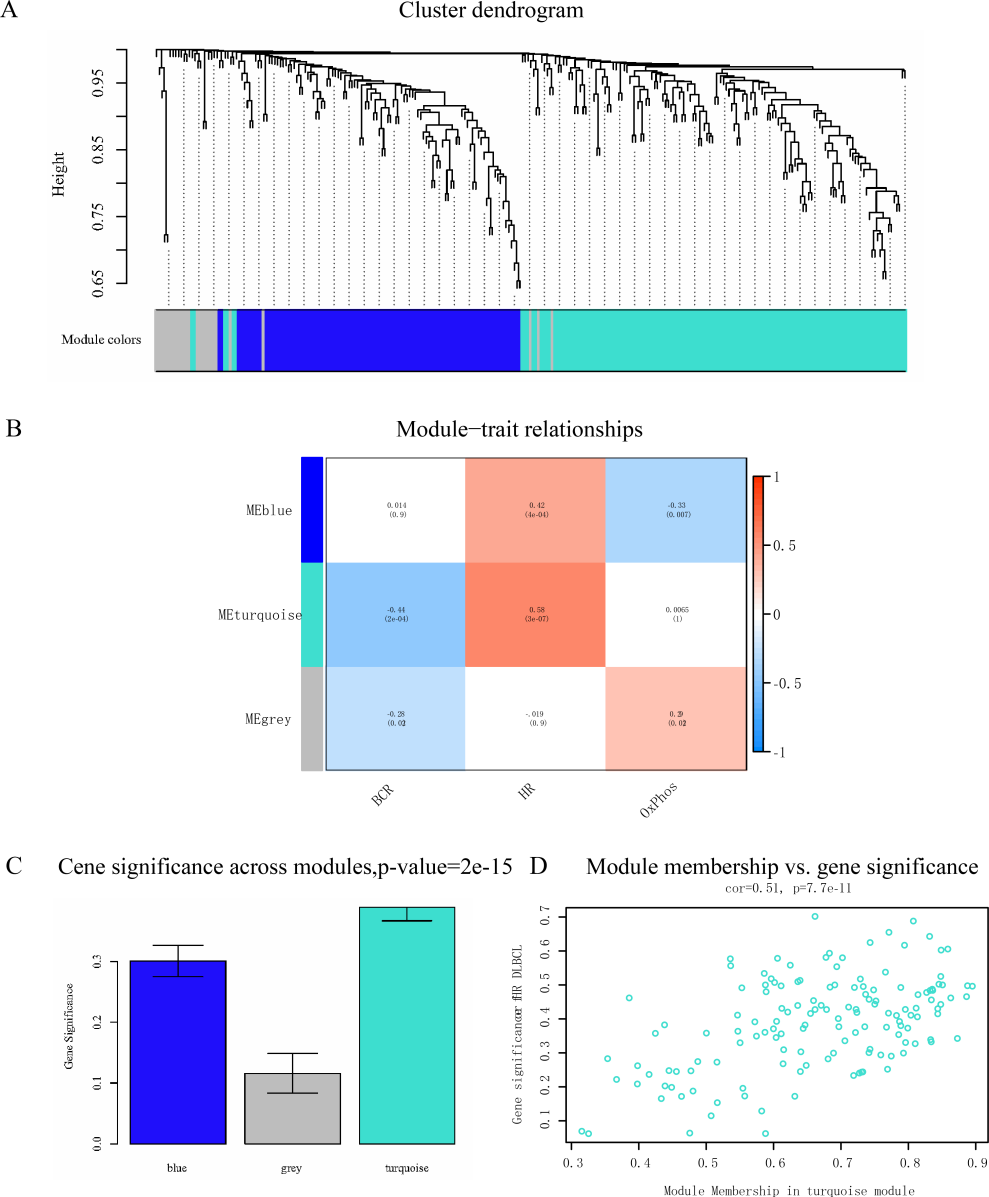
Analysis of modules correlated with the subtypes of DLBCL. (A) Dendrogram of all differentially expressed genes clustered according to a dissimilarity measure; (B) distribution of average gene significance and errors in the modules associated with DLBCL subtypes; (C) heatmap of the relevance between module eigengenes and DLBCL subtypes; (D) scatter plot of module eigengenes in the turquoise module.

After data preprocessing, the expression matrix of common DEGs was obtained in the GSE53786 training set. No sample was removed from subsequent analysis in the dataset after the first quality check (Fig. S2). To ensure a scale-free network, the power of *β* = 7 (scale-free *R*^2^ = 0.91) was selected (Fig. 3). The DEGs with similar expression patterns were clustered into modules. Three modules resulted in different colors (Fig. 4A). The grey module showed the genes that cannot be merged. According to the ME and MS, the turquoise module showed the highest correlation with HR DLBCL among all modules (Figs. 4B–4C). Moreover, Scatter plot of module eigengenes in the turquoise module was shown in Fig. 4D. Therefore turquoise module was identified as the hub module associated with HR DLBCL.

### Identification of candidate hub genes in the turquoise module

**Figure 5:**
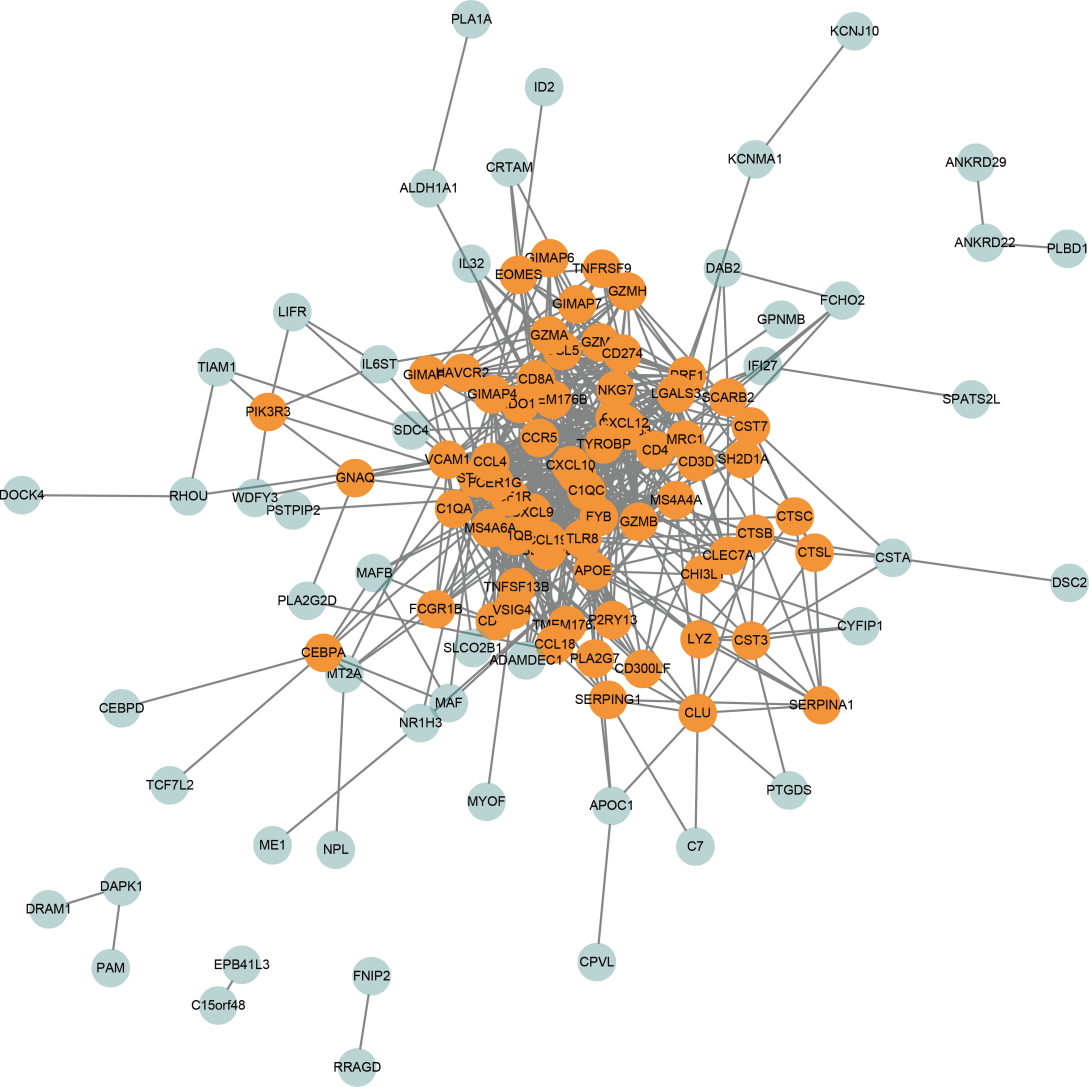
PPI network of genes in the turquoise module. The orange dots represent candidate hub genes within the turquoise module.

According to the STRING database, the PPI network for all genes in the turquoise module consisted of 119 nodes and 660 edges. Moreover, under the cutoff of node connectivity > 6, 70 genes were identified as candidate hub genes (Fig. 5). The highly connected genes in the network are considered functionally important. Therefore the 70 genes were selected for further hub gene screening.

### Hub gene screening

**Figure 6:**
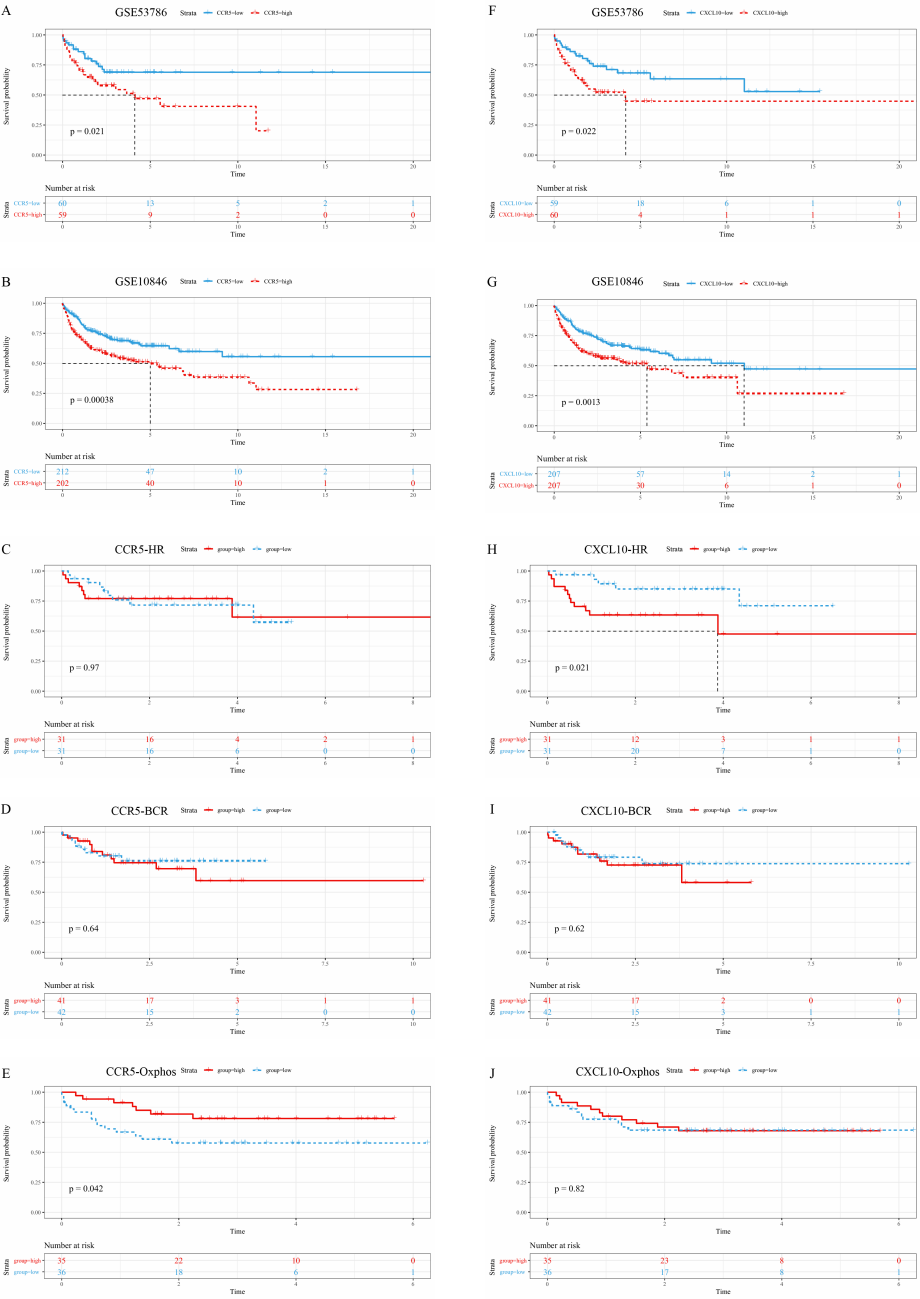
Survival analysis of hub genes. (A–B) Kaplan–Meier overall survival analysis of *CCR5* in GSE53786 and GSE10846; (C–E) Kaplan–Meier overall survival analysis of *CCR5* in the HR subtype, BCR subtype and Oxhos subtype; (F–G) Kaplan–Meier overall survival analysis of *CXCL10* in GSE53786 and GSE10846; (H–J) Kaplan–Meier overall survival analysis of *CXCL10* in HR subtype, BCR subtype and Oxhos subtype.

Kaplan–Meier survival analysis was used to determine survival with respect to candidate hub genes. Increased expression of *CCR5* and *CXCL10*, which were upregulated in the cancer group, predicted a significantly shorter overall survival (OS) (Figs. 6A–6B, Figs. 6F–6G). Next, the prognosis values of *CCR5* and *CXCL10* were assessed in different subtypes of DLBCL separately in GSE10846. Results show that high expression of CCR5 had no prognostic impact on survival in HR subtype and BCR subtype, but predicted a good prognosis in Oxphos subtype (*P* = 0.042) (Figs. 6C–6E). Besides, high expression of *CXCL10* had a significant adverse effect on the survival of HR subtype but had no significant prognostic impact on BCR subtype and Oxhos subtype (Figs. 6H–6J). Importantly, survival analysis showed that high *CXCL10* expression was associated with poor outcome in DLBCL in general, and in the HR subtype in particular. Thus, *CXCL10* was identified as the real hub gene of HR DLBCL.

### Validation of the expression level of the hub gene in DLBCL

**Figure 7:**
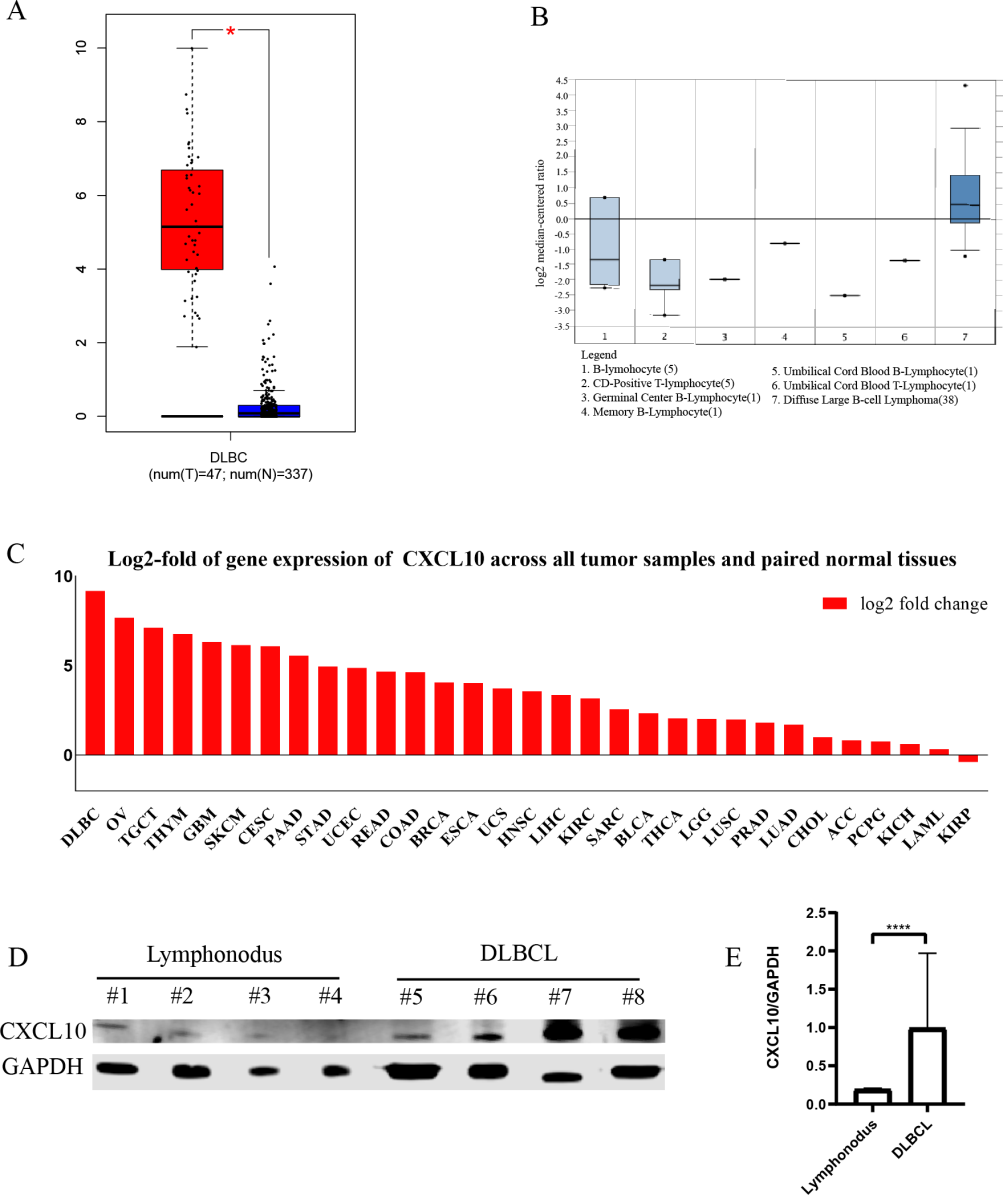
The expression of the hub gene in DLBCL. (A) The mRNA expression of *CXCL10* in DLBCL was obtained from GEPIA; (B) The mRNA expression of *CXCL10* in DLBCL was obtained from Oncomine; (C) Log2-fold of gene expression of *CXCL10* across all tumor samples vs. paired normal tissues. (D) Western blot result of *CXCL10* expression levels of lymph node samples (*n* = 4), patients with DLBCL (*n* = 4); (E) Bar chart of *CXCL10* relative protein expression (gray value of the target protein bands/gray value of the GAPDH protein bands). Mean expression ± SD is shown. (**** *P* < 0.0001, Student’s *t*-test).

Based on the GEPIA and Oncomine databases, we found that the expression of *CXCL10* was significantly elevated in DLBCL compared with normal tissues (Figs. 7A–7B). In addition, we examined the *CXCL10* expression profiles across all tumor samples and paired normal tissues. The log2-fold change in expression of *CXCL10* in DLBCL relative to that in paired normal tissues was significantly greater than that in any other type of tumor vs. normal (Fig. 7C).

The result of western blotting also revealed significantly higher expression of *CXCL10* in DLBCLs compared to lymphoid tissue (Figs. 7D–7E). Results suggested that the *CXCL10* was significantly upregulated in DLBCL and showed tumor specificity to some extent.

### Functional enrichment analysis

**Figure 8:**
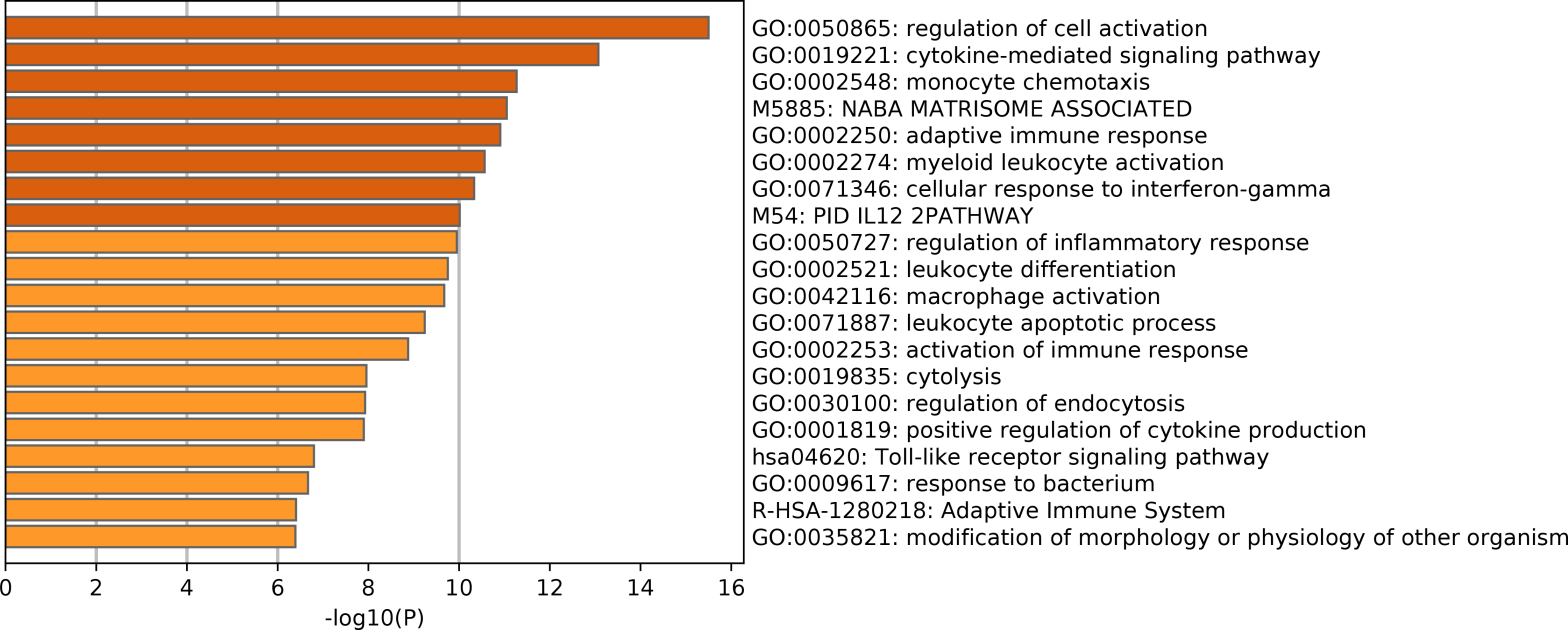
Functional and pathway enrichment analysis of DEGs of turquoise module. GO terms and KEGG pathway are presented, and each band represents one enriched term or pathway colored according to the −log 10 *P* value.

To investigate the pathogenesis of HR DLBCL, we conducted functional enrichment analysis of genes of the turquoise module according to the Metascape database. Bar graphs of the top 20 enriched terms across input gene lists, colored according to *P*-values, were shown in Fig. 8. Based on the *P*-values of these biological processes, these genes in the turquoise module were particularly enriched in regulation of cell activation, cytokine-mediated signaling pathway, monocyte chemotaxis, and other activities. The results indicate that the immune response might play a pivotal role in HR DLBCL.

### GSEA

**Figure 9:**
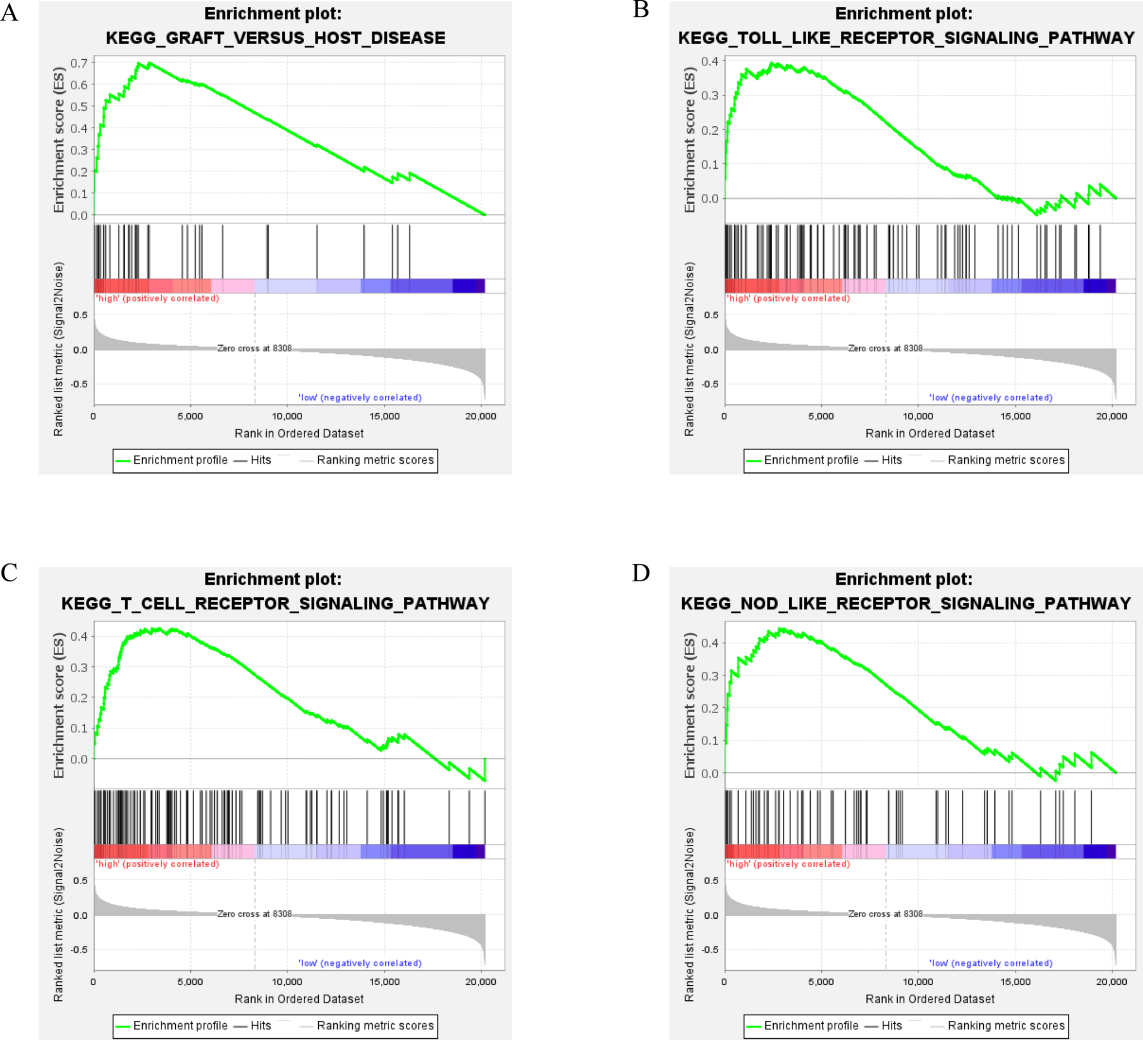
Gene set enrichment analysis in HR DLBCL. GSEA results indicating a significant correlation between the *CXCL10* expression level and the immune response, including (A) graft versus host disease, (B) Toll like receptor signaling pathway, (C) T cell receptor signaling pathway, (D) Nod like receptor signaling pathway.

To identify the potential function of the real hub gene in DLBCL, GSEA was conducted to search for KEGG pathways of the hub gene. Several immune-related pathways were enriched in the *CXCL10* highly expressed group, including “Graft versus host disease”, “Toll-like receptor signaling pathway”, “T-cell receptor signaling pathway”, “NOD-like receptor signaling pathway” was enriched in the *CXCL10* highly expressed group (Figs. 9A–9D). Therefore, *CXCL10* might be the key effector gene and have an effect on tumor immunity in HR DLBCL.

### Correlations of hub genes expression with immune infiltration level and immune makers in DLBCL

**Figure 10:**
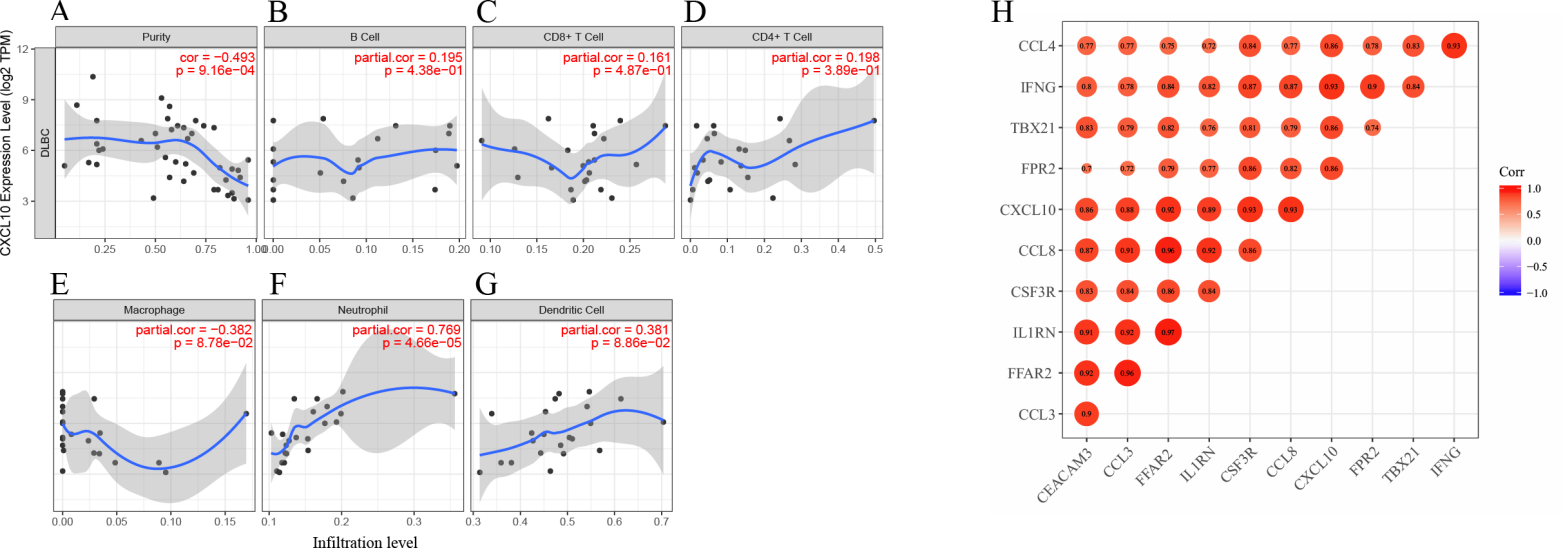
Correlations of the hub gene expression with immune microenvironment. (A–G) *CXCL10* expression level has significant correlations with tumor purity, macrophage, neutrophil, dendritic cell; (H) The correlation plot of top ten *CXCL10* correlated immune markers.

To investigate how the expression of *CXCL10* correlated with immune infiltration levels in DLBCL, we searched TIMER database. *CXCL10* expression is negatively associated with tumor purity (*r* =  − 0.493, *p* = 9.16E−04) and positively associated with the infiltration levels of neutrophil, and dendritic cell (Figs. 10A–10G). We analyzed the association between *CXCL10* expression and various immune markers further to understand the crosstalk of the hub gene with immune genes. The top 10 strongest positive correlative signatures of *CXCL10* are shown in Fig. 10H. All the relative immune signatures are listed in Table S1 (—cor—>0.3 and *p* < 0.05 is recommended).

### Construction of predicted miRNA- and TF- target regulatory networks

**Figure 11:**
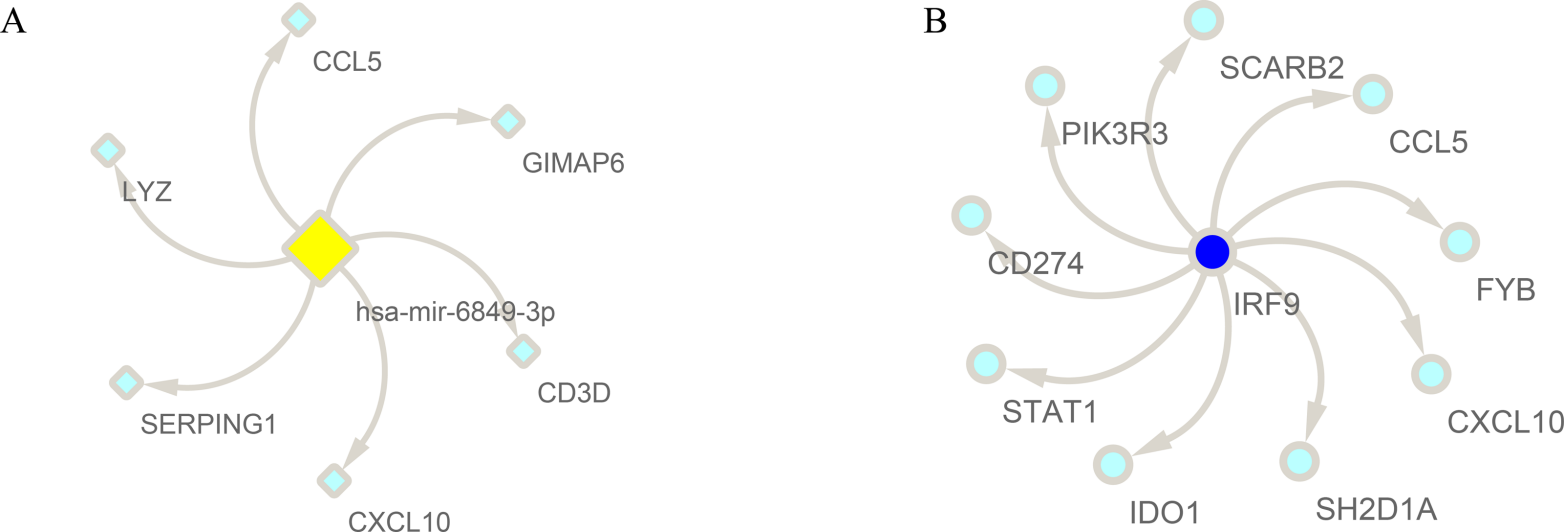
Prediction of potential regulators in the module of interest. (A) The turquoise nodes in the circle represent DEGs within the modules that regulated by hsa-mir-6849-3p; (B) the turquoise nodes in the circle represent DEGs within the modules that regulated by IRF9.

To further explore the regulation mechanisms of the hub module and hub genes, upstream regulators were predicted. A total of 630 miRNAs were predicted in the turquoise module. The network of *hsa-mir-7110-3p* with the highest degree and targeted genes in the turquoise module was displayed (Fig. 11A). The predicted TF with the highest normalized enrichment score was displayed (Fig. 11B). *IRF9* (NES = 8.140) was predicted to regulate 9 targets in the turquoise module.

## Discussion

Patients with HR DLBCL display unique clinical features, with splenomegaly and bone marrow involvement being more common. In addition, HR DLBCL cases have less frequent genetic abnormalities and occur in younger patients (Monti et al., 2005). Despite its clinical significance, the pathogenetic mechanism of HR DLBCL is poorly understood. In this study, comprehensive bioinformatic analyses of multiple datasets were used to explore the hub genes and essential pathways associated with HR DLBCL.

Disease-specific differential expression genes reveal potential biological mechanism linked to disease development. Due to the high heterogeneity of DLBCL, we performed differential genes analysis in the 3 different datasets and get overlap DEGs, to identify highly and stably expressed genes as well as lowly and stably expressed genes for further exploration of biological mechanism. Two hundred and seventy-four DEGs were identified in the three datasets. Weighted correlation network analysis was used to establish a co-expression network of common DEGs. The analysis revealed the turquoise module comprising genes significantly associated with HR DLBCL in GSE53786.

Among the genes in the turquoise module, nodes with high degree were considered functionally important. Thus 70 candidates with the highest degree of PPI network were selected. After a series of survival analyses, *CXCL10,* having significant prognosis value both in overall DLBCL patients and HR subtype, was identified as the real hub gene. These findings might contribute to the guide differential diagnosis and prognosis prediction for patients with DLBCL.

All results from the GEPIA, Oncomine databases and human tissue specimens are consistent with the findings in our study. The expression level of *CXCL10* was higher in DLBCL samples than the levels in normal tissue. According to expression profiles across all tumor samples and paired normal tissues in GEPIA, the log2-fold change in expression of *CXCL10* in DLBCL relative to that of paired normal tissues was significantly higher than that of any other type of tumor versus normal. The differential expression of *CXCL10* in DLBCL exhibited specificity to some extent. We identified that *CXCL10* is a tumor-specific gene for DLBCL. Importantly, *CXCL10* was associated with poor outcome in DLBCL, especially belonging to HR subtype. Therefore, we believe that targeting *CXCL10* may be a promising therapy with fewer side effects for DLBCL patients, especially for HR subtype.

C-X-C motif ligand 10 (*CXCL10*) is one of the chemokines commonly released by inflammatory cells (Gupta et al., 2012). Several studies (Ansell et al., 2012; Hong et al., 2017) identified the serum level of *CXCL10* as an inflammatory prognostic biomarker in patients with DLBCL. Presently, the elevated expression of *CXCL10* in DLBCL tumor tissue samples predicted disease prognosis in DLBCL. However, the exact mechanisms of *CXCL10* are yet to be reported in DLBCL. Lee et al. (2012) reported an in vivo study that monocytes promote migration and invasion of tumor cells via *CXCL10* expression in B-cell lymphoma cell lines. Natural killer (NK) cells are innate lymphocytes that are crucial in the immune response against tumor. Bernardini et al. (2017) demonstrated that NK cell compartment in MM could be modulated by the expression levels of *IP10/CXCL10*. Wendel et al. (2008) identified *CXCL10-CXCR3* signaling in NK cells as prerequisites for NK cell infiltration into tumors. The authors suggested that high levels of *CXCL10* in the tumor microenvironment represent a valuable target for therapeutic intervention by affecting myeloma NK cell surveillance.

We preliminarily explore the biological characteristics and function of the hub module and the hub gene by functional enrichment analysis and GSEA analysis. The functional enrichment analysis revealed that the DEGs in the turquoise module were markedly enriched in the regulation of cell activation, cytokine-mediated signaling pathway, monocyte chemotaxis, and other activities. GSEA analysis suggested that *CXCL10* get involved several immune-associated pathways. Of these, Toll-like receptors (TLRs) participate the regulation of immune responses to infection as innate immune receptors, and also get involved in noninfectious inflammatory diseases, like tumor invasion, survival, and tumorigenicity (Hua et al., 2009). Nucleotide-binding oligomerization domain (NOD)-like receptor signaling pathway is involved in the formation of inflammasomes, and numerous types of cancer are associated with inflamed tissue (Castano-Rodriguez et al., 2015). Both Toll-like receptors and Nod-like receptors utilize the NF-κB pathway (Van Driel et al., 2015). It has been reported that Constitutive NF-κB activation existed in DLBCL and provide advantages for proliferation and survival of these tumor cells (Compagno et al., 2009). The results have shown that the pathogenesis of HR DLBCL is closely associated with tumor immunity, and these potential mechanisms require further exploration.

Herein, the relationship between the hub gene and tumor immunity was assessed by TIMER database, which applies a deconvolution method to estimate tumor purity and the abundance of tumor-infiltrating immune cells from gene expression profiles. It was inferred that *CXCL10* is negatively associated with tumor purity and positively related to dendritic cells and neutrophil. With the increased intratumoral T cells and dendritic cells, there was a relative decrease of neoplastic B cells in HR DLBCL. Furthermore, we investigated the association of *CXCL10* with immune markers in DLBCL patients. Among the top ten immune makers correlated with *CXCL10*, chemokines *CCL3* and *CCL4* are identified as biomarkers for B cell receptor pathway activation and prognostic serum markers in DLBCL (Takahashi et al., 2015). Based on these results of the analyses, it is inferred that *CXCL10* might have a regulatory effect on tumor immunity.

Our upstream regulator analysis showed that predicted miRNA (hsa-mir-6849-3p) might regulate genes expression in the hub module. Interferon regulatory factor 9 (IRF9) , the upstream transcriptional factor of hub module, belongs to IRF family and has an established role in type I interferon responses (Sobhkhez et al., 2014).

## Conclusions

All in all, by using comprehensive bioinformatics analyses, we identified *CXCL10* as the real hub gene associated with HR DLBCL, which specific highly expressed in DLBCL and may also serve as a prognostic biomarker. And our further analysis showed that CXCL10 might have a regulatory effect on tumor immunity, which is in accordance with characteristics of immune infiltration in DLBCL. Although our results are preliminary, they provide novel insights into the molecular mechanisms of HR DLBCL. These findings will inform the development of clinically useful treatments. The tumor microenvironment is composed of non-cancerous cells and cytokines present in and around a tumor, having a major impact on the genomic analysis of tumor samples (Aran, Sirota & Butte, 2015). Tumor treatment strategies targeting both tumor cells and tumor microenvironment may contribute to HR DLBCL treatment.

##  Supplemental Information

10.7717/peerj.10269/supp-1 Supplemental Information 1 GSE44337

10.7717/peerj.10269/supp-2 Supplemental Information 2 GSE25638

10.7717/peerj.10269/supp-3 Supplemental Information 3 GSE56351

10.7717/peerj.10269/supp-4 Supplemental Information 4 GSE10846

10.7717/peerj.10269/supp-5 Supplemental Information 5 GSE53786

10.7717/peerj.10269/supp-6 Supplemental Information 6 TCGA-DLBCL

10.7717/peerj.10269/supp-7 Supplemental Information 7 Type of all the sample in GSE53786

10.7717/peerj.10269/supp-8 Supplemental Information 8 Table of immune signatures

10.7717/peerj.10269/supp-9 Supplemental Information 9 Flow diagram of dataset collection

10.7717/peerj.10269/supp-10 Supplemental Information 10 Samples clustering to detect outliers (GSE53786): no sample was above the cutline (cutHeight = 100)

10.7717/peerj.10269/supp-11 Supplemental Information 11 Heatmap of genes in the turquoise module

## Additional Information and Declarations

### Microarray Data Deposition

The following information was supplied regarding the deposition of microarray data: Data are available at NCBI GEO (GSE25638, GSE44337, GSE56315, GSE53786, GSE10846) and TCGA (TCGA-DLBCL).

### Data Availability

The following information was supplied regarding data availability: The datasets used in our study are available in the Supplementary Files.
