# Supplementary figures and images for: Identification of hub genes associated with the pathogenesis of diffuse large B-cell lymphoma subtype one characterized by host response via integrated bioinformatic analyses
Source: PeerJ. 2020 Nov 20;8:e10269. doi: 10.7717/peerj.10269 (PMC7682441; doi:10.7717/peerj.10269)

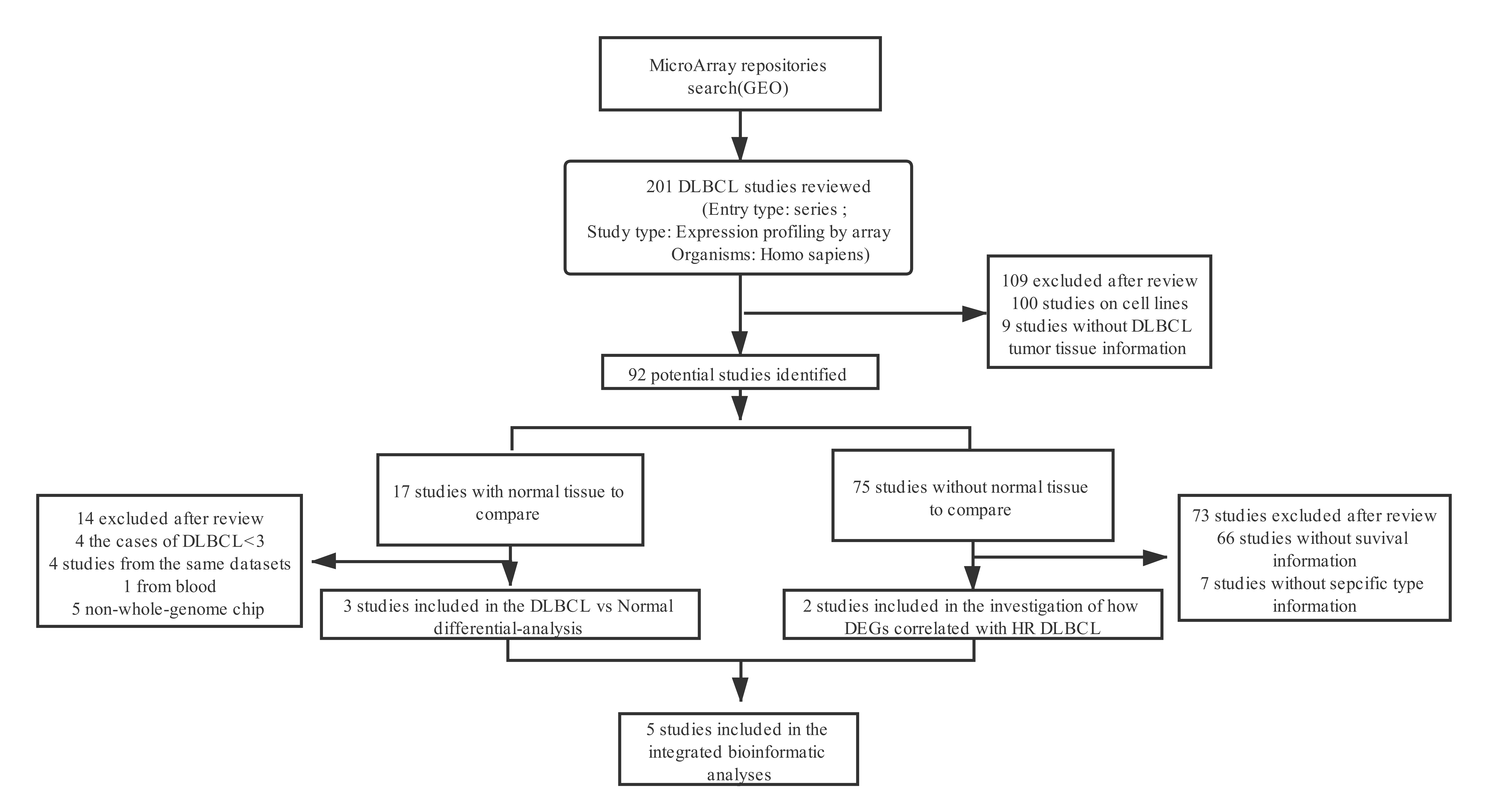

Supplement: Supplemental Information 9 [file peerj-08-10269-s009.png]

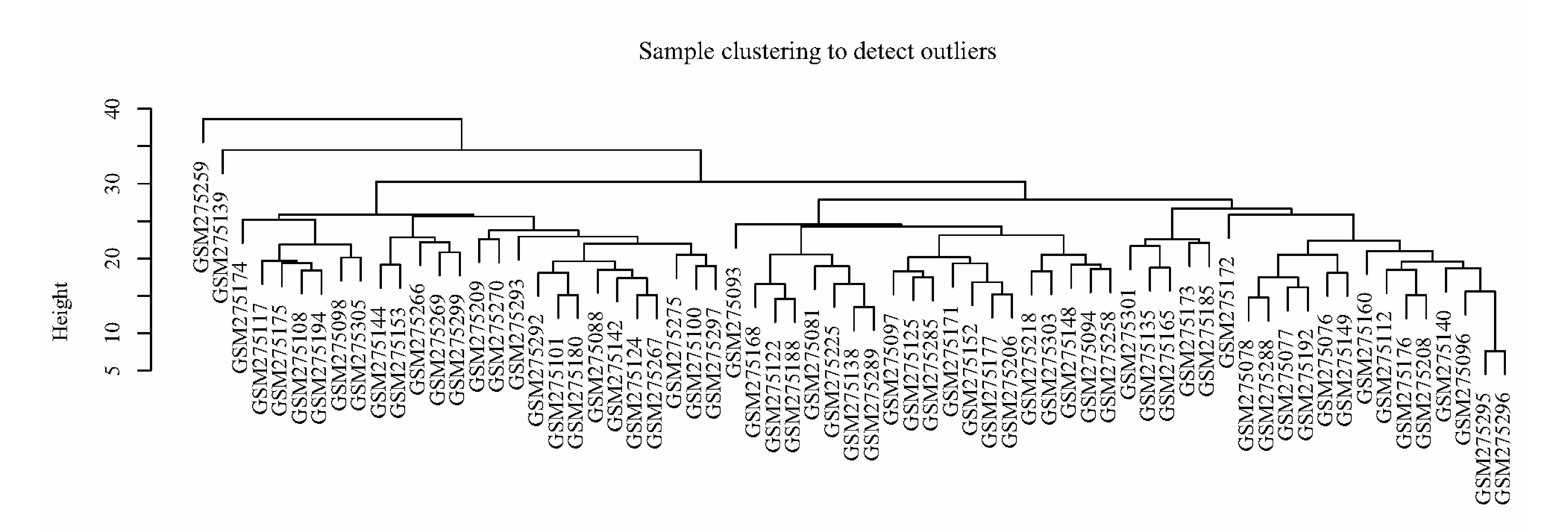

Supplement: Supplemental Information 10 [file peerj-08-10269-s010.png]

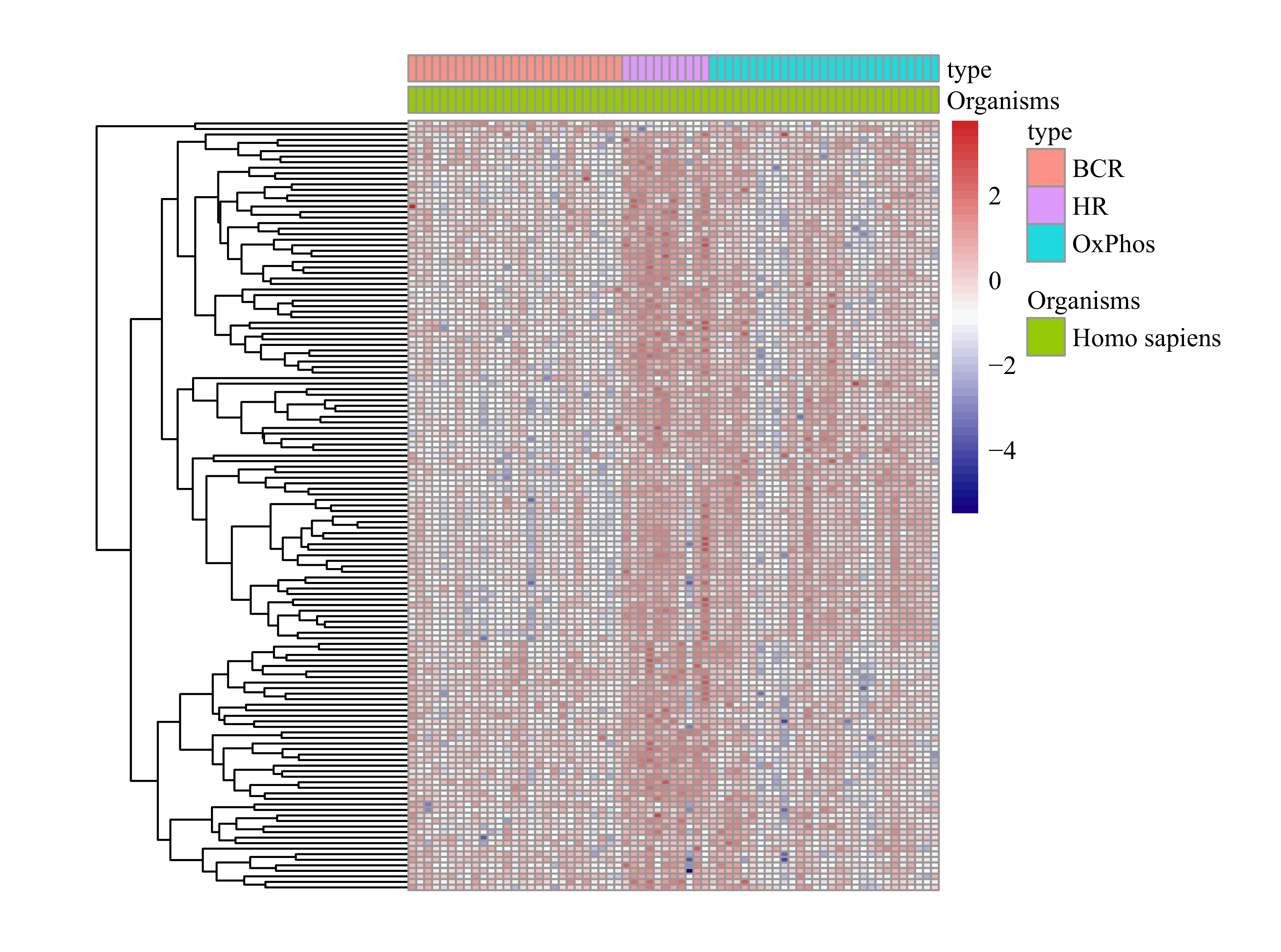

Supplement: Supplemental Information 11 [file peerj-08-10269-s011.png]
